# Sex-dependent differences in the progression of renal injury and fibrosis following ischemic acute kidney injury

**DOI:** 10.1042/CS20250136

**Published:** 2026-02-17

**Authors:** Tingfang Zhang, Zoe McArdle, Brianna K. Moore, Alyssa Di Muzio, Kate M. Denton, Robert E. Widdop, Sharon D. Ricardo

**Affiliations:** 1 Department of Pharmacology, Biomedicine Discovery Institute, Monash University, Clayton, Victoria 3800, Australia; 2 Physiology, Biomedicine Discovery Institute, Monash University, Clayton, Victoria 3800, Australia

**Keywords:** acute kidney injury, adaptive response, fibrosis, ischemia, sex differences

## Abstract

Sex differences critically influence the renal response to ischemic injury, yet the mechanisms underlying differing recovery between males and females remain incompletely understood. Using a unilateral ischemia-reperfusion model with contralateral nephrectomy (uIRIx), we performed a longitudinal analysis of the transition from acute kidney injury (AKI) to chronic kidney disease (CKD) in male and female mice following uIRIx over 98 days. Male mice developed sustained renal dysfunction, characterized by persistent proteinuria, a marked reduction in glomerular filtration rate, and progressive increases in urinary albumin/creatinine ratio, consistent with an ongoing functional decline. Histologically, males displayed extensive tubular dilation, interstitial fibrosis, and elevated kidney injury molecule-1 expression, together with persistent macrophage and T-cell infiltration indicative of unresolved inflammation. In contrast, females exhibited partial functional recovery with improved glomerular filtration rate, reduced proteinuria, and attenuated structural damage, including less fibrosis and tubular injury across all timepoints. Morphometric analysis revealed smaller glomerular cross-sectional areas in males at day 14, suggesting early maladaptive remodelling, whereas females demonstrated adaptive hypertrophy that may preserve filtration capacity. Assessment of peritubular capillaries (CD31) indicated more effective microvascular preservation in females, consistent with estrogen-mediated endothelial protection. Collectively, these findings demonstrate that females are protected from the maladaptive progression of ischemic AKI to CKD, highlighting longitudinal sex-specific dynamics in renal repair and chronic disease development.

## Introduction

Biological sex has emerged as a fundamental variable influencing the development, progression, and outcomes of kidney disease. While both males and females are susceptible to acute kidney injury (AKI), recent clinical and experimental studies indicate that males are more vulnerable to severe renal injury and experience a more rapid progression to chronic kidney disease (CKD), whereas females frequently exhibit delayed onset of pathology and are partially protected from long-term damage [1,2]. Although CKD prevalence is reportedly higher in women, more men progress to kidney failure and ultimately receive renal replacement therapy. Importantly, this pattern may reflect not only sex-specific differences in renal aging and functional decline but also differences in access to care and treatment pathways, as women may be more likely to receive or choose conservative care [3]. This paradox aligns with longitudinal evidence showing that women begin with a lower glomerular filtration rate (GFR) but experience slower age-related decline compared with men, independent of comorbidities [4,5]. Furthermore, a recent large-scale population study confirmed that eGFR trajectories differ significantly by sex, with women experiencing slower age-related decline than men, even when starting from lower baseline function [6]. These findings underscore the importance of age- and sex-specific reference values in interpreting kidney function across the lifespan.

The biological basis for these disparities is thought to be multifactorial, involving differences in sex hormones, immune responses, oxidative metabolism, and epigenetic regulation. Hormonal and immune regulation, as well as differences in oxidative stress and repair capacity, may shape sex-biased responses to kidney injury [7]. For example, females are protected from ferroptosis in proximal tubular cells due to enhanced antioxidant activity and the ovarian hormonal milieu, while males exhibit greater lipid peroxidation, inflammatory cell accumulation, and impaired repair following glutathione peroxidase deletion [8]. In parallel, sex chromosome-linked gene expression, including escape from X-chromosome inactivation and Y chromosome loss, has been shown to influence immune regulation and cellular stress responses that may contribute to sex-biased disease outcomes [9]. Multiomics analyses of mouse proximal tubules further demonstrate that sex-biased gene expression is largely driven by autosomal differences in transcription factor activity, offering new insight into cell-type-specific sex effects [10]. Moreover, it has been shown that female kidneys are protected from fibrosis and senescence in stroke-prone hypertensive rats despite aging and hypertensive stress, supporting an interplay between sex, age, and cellular injury [11].

To dissect the temporal dynamics of these sex-dependent responses, robust rodent models that represent the human AKI-to-CKD continuum are essential [12]. While the ischemia-reperfusion injury (IRI) model is widely employed to study AKI, it often fails to reproduce the chronic decline in renal function observed clinically. Incorporation of contralateral nephrectomy to unilateral IRI (uIRIx) generates a progressive model that exhibits histological and functional features of CKD, including glomerulosclerosis, tubular atrophy, interstitial fibrosis, and sustained proteinuria [13]. Sex-specific transitions from AKI to CKD were evident in male rats following IRI, which developed persistent fibrosis and proteinuria, whereas females exhibited early antioxidant responses and structural protection. However, the underlying mechanisms driving these sex differences remain incompletely defined and require further investigation through longitudinal preclinical modelling [14]. Emerging evidence also supports sex-specific patterns of glomerular remodelling and tubular repair. Denton and colleagues [11] demonstrated that female rats exhibit consistently larger glomerular cross-sectional areas with aging, potentially reflecting a compensatory hypertrophic adaptation to preserve filtration capacity. Similarly, following IRI injury, female mice showed reduced collagen deposition and epithelial-to-mesenchymal transition compared with males, associated with enhanced estrogen receptor-α signalling and protection from tubulointerstitial fibrosis [14]. These findings align with previous studies linking testosterone to increased susceptibility to apoptosis and fibrotic injury in human tubular cells [15].

Despite increasing evidence of sex differences in renal pathophysiology, few studies have systematically characterized long-term structural and functional outcomes following progressive renal injury. Here, we employed the uIRIx model to longitudinally assess male and female mice over 98 days. This approach enabled delineation of how sex and age influence renal adaptation versus maladaptation after injury. Renal function, histopathology, and injury markers, including GFR, albuminuria, fibrosis, inflammatory cell infiltration, and glomerular and tubular injury, were evaluated to determine the extent of female protection and to define the temporal and structural dynamics underlying sex-specific disease progression.

## Methods

### Animal model of AKI-CKD transition

**Figure 1:**
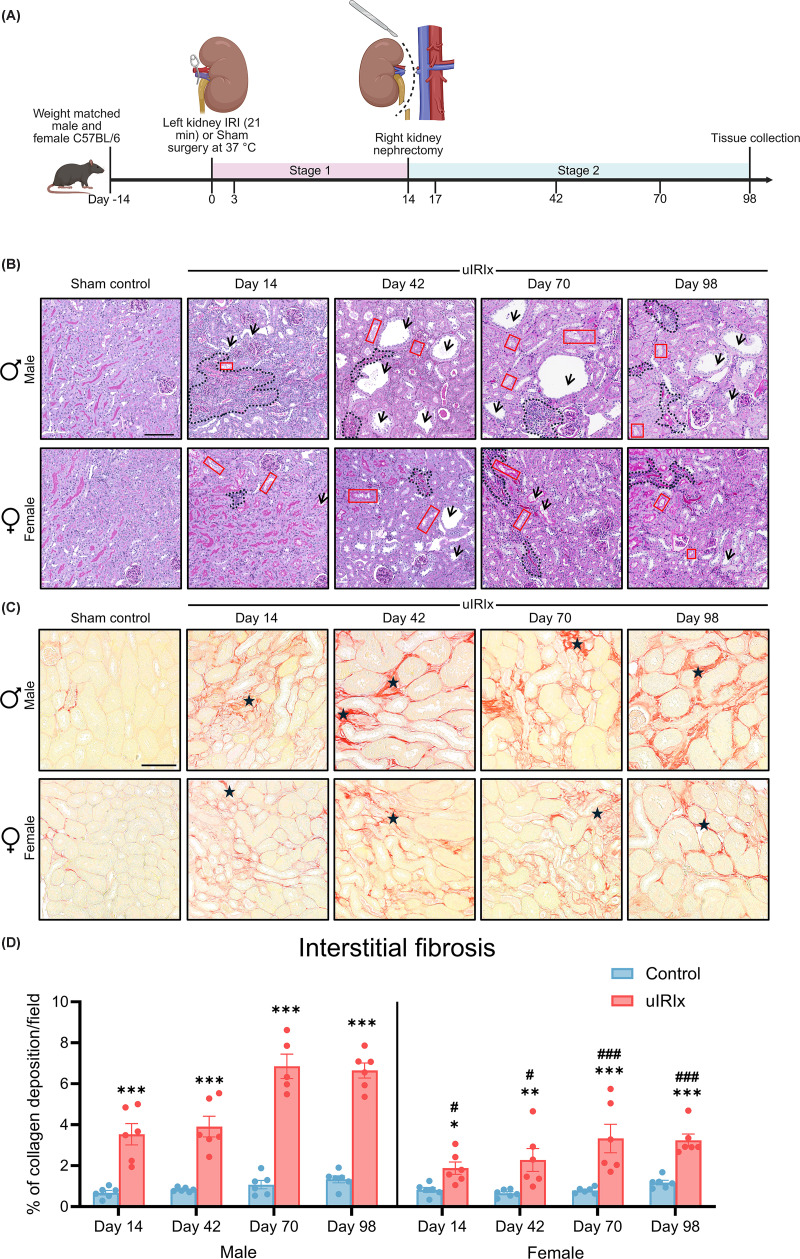
Overview of experimental design and time-dependent, sex-specific renal pathology following uIRIx (**A**) Schematic representation of the animal model and experimental timeline used in the study. (**B**) Representative images of kidneys in cortical-medullary junction stained with periodic acid Schiff (PAS) show the effects of ischemia/reperfusion injury on kidney histoarchitecture. Sham-operated kidneys displayed normal histoarchitecture with intact tubular brush borders. In contrast, uIRIx kidneys demonstrated marked tubular dilation (black arrows), loss of brush border integrity (red boxes), and interstitial expansion (dashed line). (**C**) Representative images of picrosirius red (PSR)-stained kidney sections, demonstrating interstitial collagen deposition (black star). (**D**) Quantification of interstitial fibrosis based on PSR staining, shown as the percentage of collagen-positive area relative to the total cortical-medullary field. Scale bar = 150 and 100 μm for PAS-stained and PSR-stained slides, respectively. Data are presented as mean ± standard error of the mean (SEM), *n* = 5–6. Statistical comparisons were performed using two-way ANOVA. Multiple comparison testing was performed using Tukey’s post hoc test. *P <0.05; **P <0.01; ***P <0.001 versus control at the same timepoint. ^#^*P* <0.05; ^###^*P* <0.001 versus injured male at the same timepoint.

All animal care and experiments were approved by the Monash University Animal Ethics Committee (project #39773). C57BL/6J mice were obtained from Monash Animal Services (Monash University, Clayton, Victoria, Australia) and maintained on a 12-h light/dark cycle with free access to water and Barastoc mice breeder cube (20% crude protein, 8.5% crude fat, 3.2% crude fibre, and other nutrients; Ridley, Australia), supplied as the default diet by the Monash Animal Research Platform. Buprenorphine (0.1 mg/kg) was administered subcutaneously (s.c.) to mice 30 min prior to uIRI and nephrectomy surgery prior to general anesthesia with 2%–3% inhaled isoflurane mixed with medical EP grade oxygen via a Stinger Veterinary Anesthetic machine. At the time of surgery, carprofen (5 mg/kg, s.c.) was administered and bupivacaine (0.25%) applied to the site of incision. The uIRIx mice underwent IRI surgery by clamping left kidney for 21 min at day 0, followed by a right kidney nephrectomy after 14 days [13], and mice were maintained for 98 days from the day of nephrectomy. The sham-operated control mice underwent anesthesia for the same period as IRI surgery without clamping renal pedicle. However, the right kidney was also removed after 14 days. As reported previously [13], the contralateral kidney was removed at day 14 in both IRI and control mice to impose additional physiological stress on the remaining kidney and also prevent compensatory hyperfiltration. This ensured that long-term structural and functional outcomes could be accurately assessed in the remaining index kidney. Postoperative analgesia (buprenorphine 0.1 mg/kg s.c.) was provided every 8 h for 48 h as required, and animals were monitored twice daily for signs of pain or distress. In this study, male mice were aged at 10 weeks, while female mice were aged at 12 weeks to weight-match as closely as possible at the time of surgery (Supplementary Figure S1A,B), and kidney weights were measured at the endpoint with normalized to body weight (Supplementary Figure S1C). In addition to the 98-day group, additional cohorts of mice with different endpoints include groups culled at day 14, 42, and 70 post-IR for both male and female mice (Figure 1A). At each experimental endpoint, mice were euthanized by CO_2_ asphyxiation using a gradual-fill method, with CO_2_ introduced at 20%–30% of the chamber volume per minute to reach approximately 70% CO_2_ over 2–3 min, followed by cervical dislocation as a secondary method. Group sizes (*n* = 6 per group/timepoint) were determined based on previous studies to provide sufficient reproducibility and consistency in the measured outcomes.

### Assessment of kidney function

GFR was assessed using a transdermal GFR preclinical MX (MediBeacon, St. Louis, MO, U.S.A.) in conscious mice, as previously described [16] with protocol optimizations. Mice were depilated prior to measurement, and a fluorescence detector was secured to the depilated skin. FITC-sinistrin (7.5 mg/100 g body weight) was administered via tail vein under brief isoflurane anesthesia, after which mice were returned to individual cages for a 90-min recording period. Devices were then removed, raw data were read using MB Lab (MediBeacon), and estimated GFR was calculated using MB studio (MediBeacon) based on the tracer’s clearance kinetics [17]. Urine was collected using metabolic cages for 24 h, and the urinary albumin level was determined using a mouse albumin ELISA kit (Crystal Chem #80630, Elk Grove Village, IL, U.S.A.) according to the manufacturer’s protocol. Urine osmolality was measured in duplicates using the OsmoPRO Multi-Sample Micro-Osmometer (Advanced Instruments, Norwood, MA, U.S.A.) and urinary creatinine level was measured using the creatinine assay kit (ab65430; Abcam, Cambridge, U.K.), and urinary albumin/creatinine ratio (uACR) was calculated.

### Histological staining and quantitative assessment

Fixed kidneys were processed and embedded in paraffin wax and sectioned at 4 μm thickness. Serial sections were stained with either PAS or PSR for assessment of accumulation of interstitial collagen type I and III. For quantification of glomerulosclerosis, 15 glomeruli were randomly selected in each PAS-stained kidney section per animal for scoring. Scoring was performed using Aperio ImageScope software (Leica Biosystems, Nussloch, Germany) in conjunction with a representative scoring criterion that evaluated glomerular architecture, immune cell infiltration, extracellular matrix accumulation, and capillary integrity. Glomerulosclerosis severity was graded on a scale from 0 (no sclerosis, 0% sclerotic area) to 5 (global sclerosis, 100% sclerotic area).

Tubular dilation was also assessed in PAS-stained kidney sections, where six images were randomly selected from cortical regions. The percentage area of white space was measured for each image as an indicator of tubular dilation. Glomerular hypertrophy was also assessed in PAS-stained sections by manually tracing individual glomerular tufts using Aperio ImageScope. Thirty glomeruli per section were selected based on two inclusion criteria: a clearly defined boundary between the glomerular tuft and Bowman’s capsule, and the presence of a vascular pole, indicating mid-glomerular sectioning for accurate area estimation as previously published [11].

For quantification of the PSR-stained slides, five consecutive non-overlapping fields of the cortico-medullary junction were captured and randomly analyzed to semi-quantify collagen type I and III accumulation at 200× magnification. The results were expressed as the percentage of stained areas relative to the total viewed field. The PAS-stained kidney sections were scanned using the Aperio Scanscope AT Turbo brightfield scanner (Leica Biosystems, Nussloch, Germany). PSR-stained and immunohistochemistry-stained slides were imaged using an Olympus PhoenIX-81 microscope (Olympus Corporation, Tokyo, Japan). All image analyses were performed in a blinded manner and using ImageJ software (version 1.52n, National Institutes of Health, Bethesda, MD, U.S.A.).

### Immunohistochemistry staining

Paraffin-embedded kidney sections were cut at 4 μm thickness and processed for immunohistochemical detection of kidney injury molecule-1 (KIM-1), CD31, CD3, and F4/80. Antigen retrieval was performed in DAKO citrate buffer at 95°C for 30 min. Endogenous peroxidase activity was quenched with 3% hydrogen peroxide for 10 min. For KIM-1 staining, non-specific binding was blocked using an Avidin/Biotin Blocking Kit (SP-2001, Vector Laboratories, Burlingame, CA, U.S.A.), followed by incubation with blocking serum for 1 h at room temperature. Sections were then incubated overnight at 4°C with a rat anti-mouse monoclonal (IgG_2B_) antibody, clone #222414, to detect KIM-1 (MAB1817, 1:500 dilution; R&D Systems, Minneapolis, MN, U.S.A.). The following day, the biotinylated secondary antibody was applied using the ECTASTAIN Elite ABC-HRP Kit (PK-6102, Vector Laboratories) according to the manufacturer’s protocol. Immunoreactivity was visualized using DAB chromogen (Dako, Cat# K3468, Agilent Technologies, Santa Clara, CA, U.S.A.) and counterstained with hematoxylin (Amber Scientific, WA, Australia).

For CD31, CD3, and F4/80 staining, sections were blocked with Dako Protein Block Serum-Free (X0909, Dako) and incubated overnight at 4°C with primary rabbit anti-human polyclonal (IgG) CD31 antibody (ab28364, 1:250 dilution; Abcam, Cambridge, U.K.), CD3 (ab16669, rabbit anti-mouse monoclonal; clone #SP7, 1:200; Abcam), or F4/80 (#70076, rabbit anti-mouse monoclonal; clone D2S9R, 1:500; Abcam). Detection was carried out using the EnVision+ System-HRP (DAKO, Cat# K4003, Agilent Technologies). according to the manufacturer’s instructions. Slides were counterstained with hematoxylin, dehydrated, and mounted. All stained sections were imaged on an Olympus PhoenIX-81 microscope using identical exposure and gain settings. Blinded image analysis was performed using ImageJ software using a fixed threshold determined from control slides and applied uniformly to all images for each marker.

### Statistical analysis

All data are expressed as the means ± SEM, and statistical analysis was performed using GraphPad Prism software v10.5.0 (GraphPad Software Inc., San Diego, CA, U.S.A.). Two-way ANOVA was used for most datasets in this study, applied separately to evaluate (i) sex- and time-dependent effects within either control or injured groups, (ii) sex- and injury-dependent differences at individual timepoints, and (iii) injury- and time-dependent effects within each sex group with a Tukey’s post hoc test. Glomerulosclerosis scoring data were analyzed using the non-parametric Mann–Whitney U test for between-group comparisons at each timepoint. A *P*-value <0.05 was considered statistically significant.

## Results

### Time-dependent sex differences in histopathology in the kidneys of the mice following uIRIx

To establish a model of progressive kidney injury, male and female mice underwent uIRIx, with tissue collection up to day 98 (Figure 1A). Kidney weight normalized to body weight differed between uIRx and sham kidneys at both day 14 and day 98 endpoints in male mice, whereas no significant differences were observed in female mice at all timepoints (Supplementary Figure S1C). Histopathology of the kidneys of sham-operated controls from both male and female mice showed normal cortico-medullary architecture, with intact proximal tubular brush borders and only a small number of resident interstitial inflammatory cells (Figure 1B). However, kidney sections from injured male mice revealed persistent tubular injury, evident as early as day 14 post-IRI. Injured male kidneys exhibited extensive tubular dilation, cast formation, interstitial immune cell infiltration, and disruption of the brush border. These features increased in severity over time, with persistent architectural disorganization and interstitial expansion observed through day 98 (Figure 1B). In contrast, female kidneys maintained relatively preserved tubular morphology with more intact epithelial lining. Although mild dilation and brush border attenuation were observed in some regions, these changes were generally less pronounced across time.

### Time-dependent sex differences in collagen I and III accumulation in uIRIx kidneys

The intensity and distribution of PSR staining was used to quantify the accumulation of interstitial type I and III collagen. In male mice that underwent sham surgery (Figure 1C), only a normal minimal amount of collagen accumulation was evident, representing the delicate framework of connective tissue within the kidney, although a gradual age-related increase was observed across timepoints (Figure 1D). Collagen deposition in uIRIx male kidneys was consistently higher than in sham-operated controls at each examined timepoint (day 14: 3.53 ± 0.52%, day 42: 3.91 ± 0.50%, day 70: 6.85 ± 0.59%, and day 98: 6.64 ± 0.37%), with progressive accumulation observed from day 14 to day 70 (*P* <0.0001). Beyond day 70, collagen levels remained elevated but did not increase further by day 98.

In female mice, interstitial collagen deposition in the sham-operated kidneys remained significantly lower than that in injured mice across all timepoints (Figure 1D), consistent with preserved tissue architecture. Following uIRIx, injured female kidneys exhibited a significant increase in collagen content relative to their respective sham controls at each timepoint (day 14: 1.88 ± 0.30%, day 42: 2.28 ± 0.56%, day 70: 3.33 ± 0.70%, and day 98: 3.24 ± 0.30%). At all corresponding timepoints, collagen deposition in injured female kidneys remained lower than that observed in injured male kidneys, indicating a sex-dependent attenuation of interstitial fibrosis. A time-dependent trend toward greater interstitial fibrosis was also observed (*P* <0.05), with collagen accumulation gradually rising from day 14 to day 70, after which levels plateaued with no further increase detected by day 98 (Figure 1D).

### Temporal progression of kidney function after uIRIx in male and female mice

**Figure 2:**
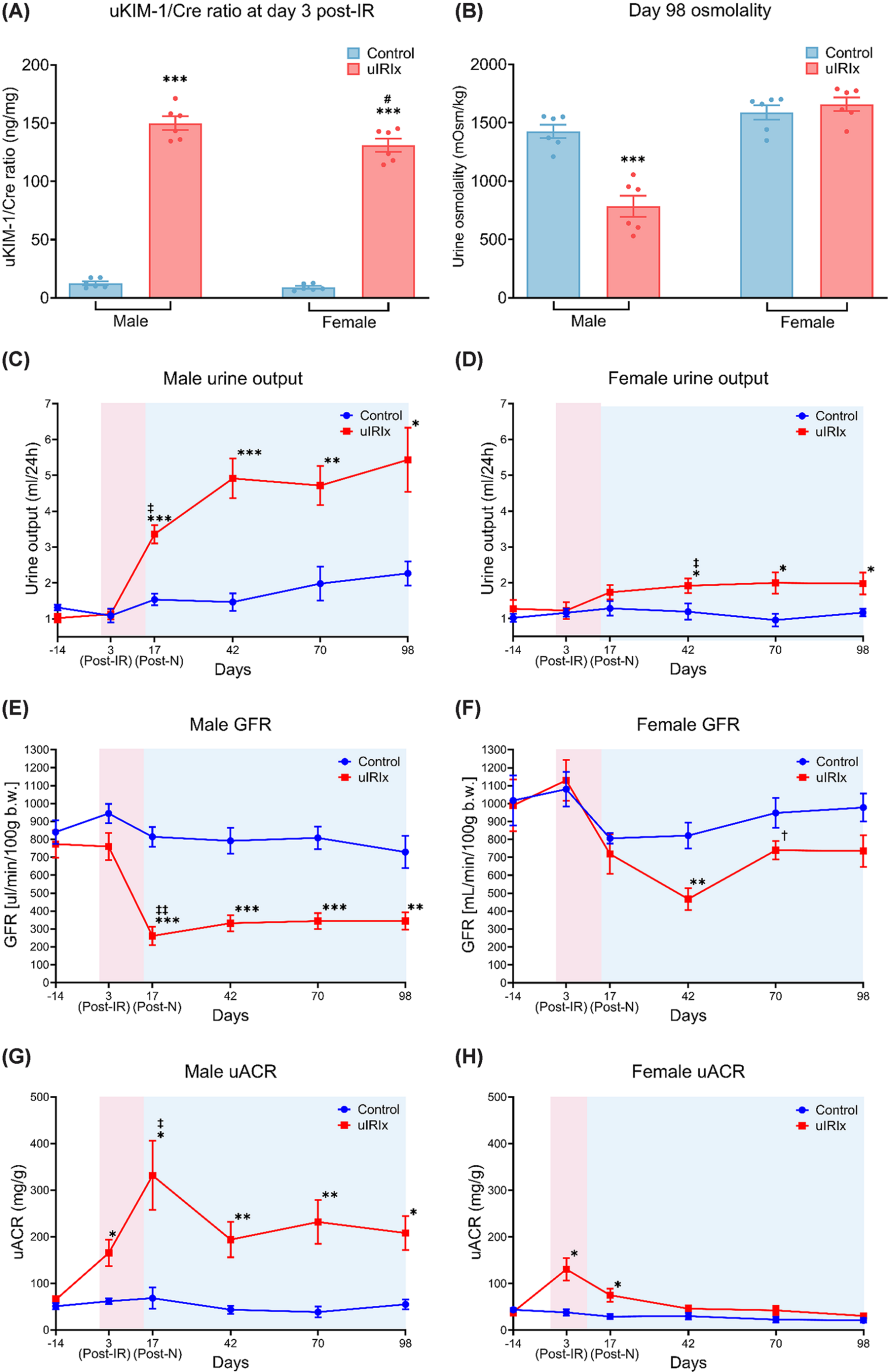
Time-course analysis of kidney function following uIRIx in male and female mice (**A**) uKIM-1/Cre ratio was measured on day 3 post-IR to indicate the severity of acute tubular injury. (**B**) Urine osmolality was measured on day 98 post-IR to assess kidney concentrating ability. (**C**,** D**) Twenty-four-hour urine output measured in metabolic cages at baseline (day 14) and at post-injury timepoints in male and female mice. (**E, F**) GFR demonstrating sustained impairment in male IRI mice with partial recovery in females. (**G, H**) uACR, representing glomerular barrier function, increased significantly after injury and remained elevated in males, whereas females showed a transient increase followed by resolution. Data are presented as mean ± SEM, *n* = 5–6. Statistical comparisons were performed using a mixed-effects model with Tukey’s post hoc test, accounting for repeated measures and occasional missing values. *P <0.05; **P <0.01; ***P <0.001 versus control within the sex at the same timepoint. #*P* <0.05; ^‡^*P* <0.05; ^‡‡^P <0.01 versus day -14 baseline measurement. ^†^*P* <0.05 versus day 42.

Renal function was assessed over time in the same cohort of mice for up to 98 days following uIRIx (Figure 2). At day 3, post-IR (Figure 2A), the uKIM-1/Cre ratio was significantly elevated in both male (149.94 ± 5.88 ng/mg versus 12.56 ± 1.62 ng/mg, *P* <0.001) and female (131.01 ± 5.68 ng/mg versus 8.93 ± 1.14 ng/mg, *P* <0.001) mice compared with their respective controls. However, injured male mice also exhibited significantly higher levels than injured females (*P* <0.05), suggesting more severe tubular injury in male mice in response to IRI. By day 98, urine osmolality was significantly (*P* <0.001) reduced in injured male mice relative to controls (Figure 2B). This reduction in concentrating ability is consistent with persistent tubular dysfunction and aligns with the significantly lower GFR observed in males at the same period, suggesting progressive CKD and impaired renal reserve. In contrast, injured females displayed urine osmolality values similar to controls at day 98.

Injured male mice exhibited significantly higher 24-h urine output than sham-operated controls at all timepoints post-IR (Figure 2C), reflecting persistent polyuria and impaired tubular concentrating ability following uIRIx. In contrast, female mice showed a delayed increase in urine output, with significant polyuria emerging only from day 42 onwards relative to baseline (Figure 2D). This delayed and less pronounced polyuric response in females was consistent with their comparatively preserved tubular function early after injury.

Moreover, GFR assessed by transcutaneous FITC-sinistrin with representative clearance curves provided in Supplementary Figure S2 declined markedly in male mice following uIRIx. Compared with their baseline (773.70 ± 76.33 μl/min/100 g) measured at day 14, GFR fell significantly post-nephrectomy onwards (261.01 ± 51.18 μl/min/100 g at day 17; ∼67.9% reduction) and remained persistently reduced until day 98 (day 42: 332.04 ± 44.55 μl/min/100 g; day 70: 344.28 ± 44.39 μl/min/100 g; day 98: 344.78 ± 49.29 μl/min/100 g), with no evidence of recovery (Figure 2E). Additionally, uIRIx male mice also exhibited significantly lower GFR than sham-operated controls at all post-nephrectomy timepoints (day 17: 814.18 ± 54.76 μl/min/100 g; day 42: 791.76 ± 72.22 μl/min/100 g; day 70: 808.10 ± 63.68 μl/min/100 g; day 98: 729.25 ± 89.44 μl/min/100 g). In contrast, female mice experienced a moderate ∼43.0% reduction in GFR following uIRIx injury (Figure 2F). Although GFR declined significantly in uIRIx mice at day 42 compared with sham-operated mice (467.80 ± 61.85 μl/min/100 g versus 821.41 ± 72.42 μl/min/100 g, *P* <0.01), partial recovery was observed at later stages in females, with GFR improving by day 70 (739.55 ± 51.84 μl/min/100 g) and remaining stable through day 98 (735.72 ± 88.71 μl/min/100 g) (Figure 2F), and the significant difference between injured mice and sham-operated mice was no longer evident at day 70 and day 98.

In male mice (Figure 2G), uACR increased progressively following IRI (*P* <0.05), peaking at day 17 post-nephrectomy, and was significantly increased compared with sham-operated control mice (*P* <0.05). Although uACR declined slightly thereafter, it remained significantly elevated from day 42 onwards compared with sham-operated controls (day 42: 193.93 ± 37.85 mg/g versus 43.65 ± 8.60 mg/g, *P* <0.05; day 70: 231.77 ± 47.19 mg/g versus 38.64 ± 11.710 mg/g, *P* <0.01; day 98: 208.22 ± 36.48 mg/g versus 55.03 ± 10.57 mg/g, *P* <0.05). In contrast, female mice exhibited only a modest and transient elevation in uACR following injury, with a small but statistically significant elevation at day 3 and day 17 compared with sham-operated controls (day 3: 130.60 ± 24.26 mg/g versus 38.16 ± 7.18 mg/g, *P* <0.01; day 17: 75.10 ± 13.81 mg/g versus 29.29 ± 5.69 mg/g, *P* <0.05). However, from day 42 onward, uACR values returned to near-baseline levels and remained low thereafter (Figure 2H).

### Time-dependent sex differences in the progression of glomerular hypertrophy and sclerosis

**Figure 3:**
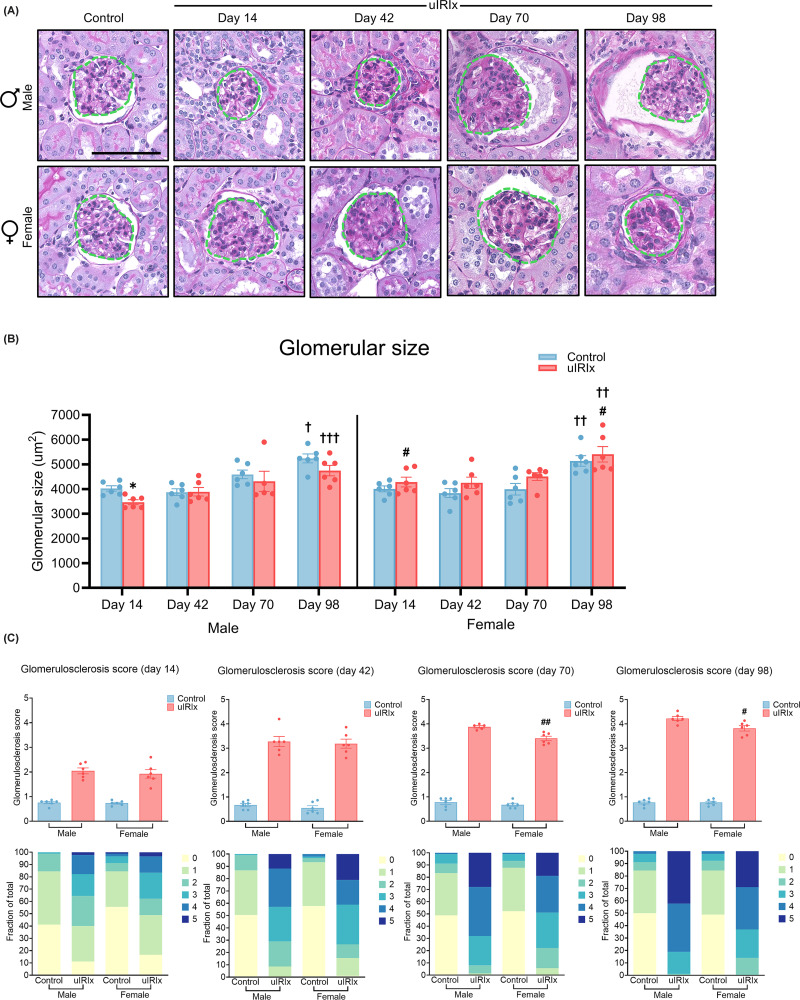
Sex-specific glomerular changes following uIRIx (**A**) Representative PAS-stained kidney sections from male and female mice at indicated timepoints after uIRIx (green dash line indicates measurement of glomerular hypertrophy). Scale bars = 100 μm. (**B**) Quantification of glomerular cross-sectional area showing temporal changes in glomerular hypertrophy after injury. Data are mean ± SEM, *n* = 5–6 per group. Two-way ANOVA with Tukey’s post-hoc test was used for statistical comparison. (**C**) Glomerulosclerosis scores (top) and corresponding distribution of individual glomerular grades (bottom) for each timepoint in male and female mice. Groups were compared using the non-parametric Mann–Whitney test. *P <0.05 versus control within the sex at the same timepoint. ^#^*P* <0.05; ^##^*P* <0.01 versus injured male at the same timepoint. ^†^*P* <0.05; ^††^*P* <0.01; ^†††^*P* <0.001 versus the same group within the same sex at day 14.

Glomerular size was measured to investigate injury-related compensatory mechanisms of glomerular remodelling after injury (Figure 3A). A gradual increase in glomerular size was observed in both male and female mice from day 14 (male control: 4025.26 ± 106.43 μm^2^, male uIRIx: 3468.11 ± 97.03 μm^2^, female control: 4006.53 ± 121.16 μm^2^, and female uIRIx: 4287.35 ± 198.19 μm^2^) to day 98 (male control: 5239 ± 181.26 μm^2^, male uIRIx: 4745.57 ± 212.89 μm^2^, female control: 5139.04 ± 213.66 μm^2^, and female uIRIx: 5408.23 ± 311.14 μm^2^), with statistically significant differences evident between day 14 and day 98 regardless of injury or not, supporting progressive age-associated hypertrophy in both sexes, or compensatory hypertrophy due to nephrectomy. At day 14, glomerular size in uIRIx male mice was significantly smaller than that in both sham-operated male controls (*P* <0.5; Figure 3B) and female mice subjected to the same injury (*P* <0.5; Figure 3B). This difference was not significant at day 42 or 70, during which glomerular sizes between injured males and females were comparable. However, by day 98, glomerular size in uIRIx male mice again appeared significantly smaller than that of female counterparts (*P* <0.5; Figure 3B). The distribution of glomerular injury severity, as shown in the heatmaps (Figure 3C), further demonstrated a higher proportion of severely sclerotic glomeruli (grades 4–5) in uIRIx male mice, especially at later stages. In contrast, uIRIx female mice exhibited a predominance of mildly affected glomeruli (grades 2–4), with fewer reaching advanced sclerosis scores (Figure 3C).

### Time-dependent sex differences in the progression of tubular injury following uIRIx

**Figure 4:**
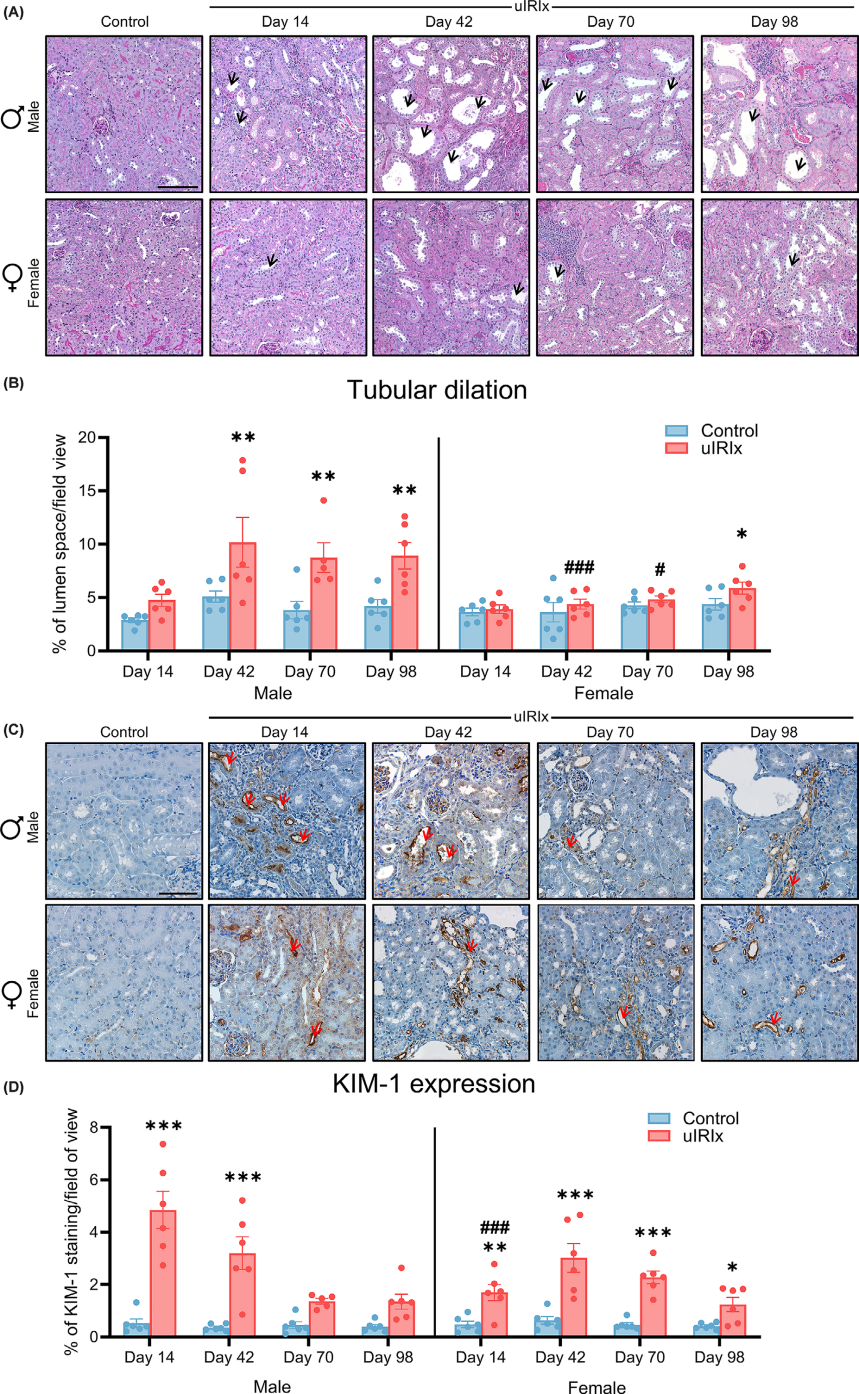
Time-course analysis of tubular dilation and KIM-1 expression following uIRIx in male and female mice (**A**) Representative PAS-stained kidney sections from male and female mice showing progressive tubular dilation in male uIRIx kidneys. (**B**) Quantification of tubular dilation (black arrows), expressed as the percentage of lumen space per field of view. (**C**) Representative immunohistochemical staining of KIM-1 (red arrows) in proximal tubules, and (**D**) quantification of KIM-1 expression, shown as percentage of KIM-1-positive area per field. KIM-1 expression was highest at early timepoints and declined over time. Scale bar = 150 μm and 100 μm for PAS-stained and immunohistochemistry (KIM-1) staining, respectively. Data are presented as mean ± SEM, *n* = 5–6. Statistical comparisons were performed using two-way ANOVA and multiple comparison testing was performed using Tukey’s post hoc test. *P <0.05; **P <0.01; ***P <0.01 versus control within the sex at the same timepoint. ^#^*P* <0.05; ^###^*P* <0.001 versus injured male at the same timepoint.

Tubular injury was assessed by histological evaluation of tubular dilation (Figure 4A,B) and by immunohistochemistry staining for KIM-1, a proximal tubular injury marker (Figure 4C,D). In male mice, tubular dilation was evident as early as day 14, characterized by prominent widening of tubular lumens in the cortico-medullary region. Quantification of tubular dilation, performed by calculating the proportion of luminal space within the cortical-medullary area, showed a significant elevation from day 42 (*P* <0.01) onwards compared with sham-operated control mice, which persisted through day 98 (*P* <0.01). In female mice, a significant increase in tubular dilation was only observed 98-day post-injury compared with female sham-operated control mice. At day 42 and 70, uIRIx males exhibited significantly more tubular dilation than injured females (day 42: 10.18 ± 2.33% versus 3.62 ± 0.90%, *P* <0.001; day 70: 8.75 ± 1.38% versus 4.83 ± 0.31%, *P* <0.05).

KIM-1 expression was minimal in sham-operated mice of both sexes across all timepoints (Figure 4D). Injured male mice exhibited a significant increase in KIM-1 expression at day 14 compared with sham-operated controls (*P* <0.001). Although KIM-1 levels gradually declined over time, the expression on day 42 remained significantly elevated (*P* <0.001). At later timepoints (day 70 and 98), KIM-1 levels in injured males were not statistically significant compared with controls. In comparison, injured female mice also showed increased KIM-1 expression following uIRIx. A moderate but significant elevation was observed at day 14 (*P* <0.01), which peaked at day 42 (*P* <0.001). Notably, KIM-1 expression in injured females at day 14 was significantly lower than that observed in injured males at the same timepoint (*P* <0.001). Although expression levels remained higher than in sham controls at later timepoints, the magnitude of increase similarly exhibited a declining trend over time, as observed in males.

### Time-dependent sex differences in the inflammatory responses following uIRIx

**Figure 5:**
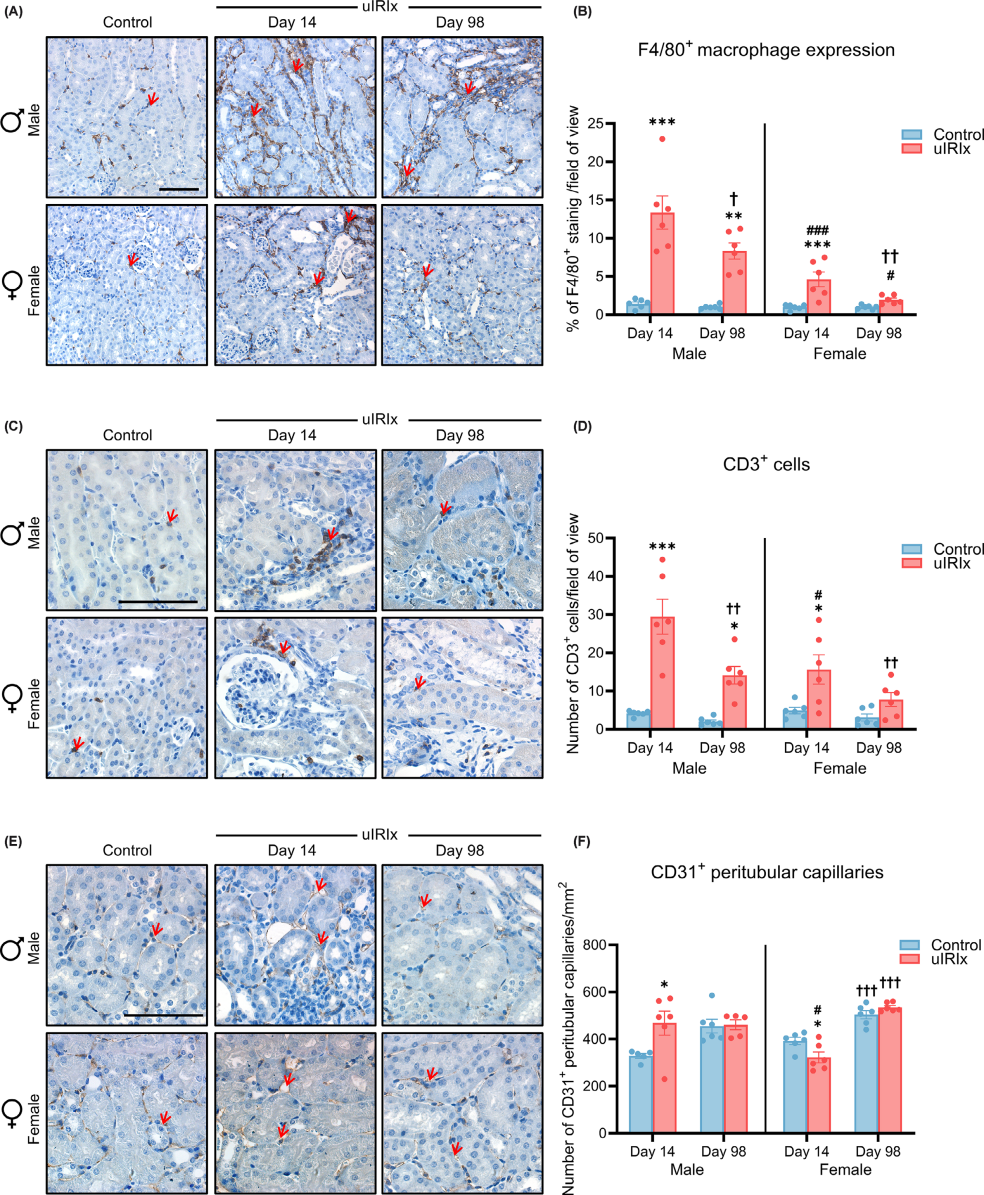
Inflammatory cell infiltration and peritubular capillary loss following uIRIx (**A**) Representative F4/80 immunostaining and (**B**) quantification showing macrophage accumulation in male and female kidneys at days 14 and 98 after uIRIx. (**C**) Representative CD3 immunostaining and (**D**) quantification of T cell infiltration at corresponding timepoints. (**E**) Representative CD31 immunostaining and (**F**) quantification of peritubular capillary density demonstrating capillary rarefaction after injury. Red arrows indicate DAB positive staining. Scale bar = 100 μm. Data are mean ± SEM, *n* = 5–6. Statistical comparisons were performed using two-way ANOVA, and multiple comparison testing was performed using Tukey’s post hoc test. *P <0.05; **P <0.01; ***P <0.001 versus control within the sex at the same timepoint. ^#^*P* <0.05; ^###^*P* <0.001 versus injured male at the same timepoint. ^†^*P* <0.05; ^††^*P* <0.01; ^†††^*P* <0.001 versus the same group within the same sex at day 14.

Inflammatory cell infiltration was assessed by immunohistochemistry for F4/80^+^ macrophages and CD3^+^ T cells (Figure 5A–D). In male mice, F4/80^+^ macrophages were markedly increased at day 14 following uIRI and remained elevated at day 98 compared with sham controls (day 14: 13.37 ± 2.17% versus 1.44 ± 0.24%; *P* <0.001; day 98: 8.33 ± 1.05% versus 1.01 ± 0.12%; *P* <0.01). Moreover, F4/80 expression at day 98 was significantly reduced compared with day 14 (*P* <0.05) within the uIRIx group, indicating a partial resolution of macrophage infiltration over time. In female mice, macrophage infiltration showed a declining trend over time. At day 14, F4/80^+^ macrophages were significantly increased compared with sham controls (*P* <0.001), whereas by day 98 the levels had returned to baseline and were not different from sham controls. Importantly, at both timepoints female uIRIx mice exhibited significantly lower F4/80^+^ cell infiltration than male uIRIx counterparts (day 14: *P* <0.001; day 98: *P* <0.05).

Similarly, in male mice, CD3^+^ T cells were markedly increased at day 14 following uIRI and remained elevated at day 98 compared with sham controls (day 14: *P* <0.001; day 98: *P* <0.05). A significant decline was also observed between day 14 and day 98 within the uIRIx group (*P* <0.01). In female mice, CD3^+^ infiltration was elevated after uIRIx, with a significant increase observed at day 14 compared with sham controls (*P* <0.05), and by day 98 the levels had declined and were not different from sham controls. When comparing sexes, male uIRIx mice exhibited significantly greater CD3^+^ infiltration than females at day 14 (*P* <0.05), whereas no significant difference was detected at day 98.

### Time-dependent sex differences in peritubular capillary rarefaction following uIRIx

Peritubular capillaries were evaluated by CD31 immunostaining (Figure 5E,F). At day 14, male and female mice exhibited opposing vascular responses to uIRI. In males, CD31^+^ capillary density was significantly increased compared with sham controls (*P* <0.05), whereas in females a significant decrease was observed relative to controls (*P* <0.05). From day 14 to day 98, both sham-operated and uIRIx female groups showed a significant increase in CD31^+^ capillary density over time (*P* <0.001). However, when compared with their respective sham controls at day 98, both male and female uIRIx mice did not display significant differences.

## Discussion

This study reveals sex-dependent differences in kidney structure and function following ischemic injury using a clinically relevant uIRIx model that mimics the transition from AKI to CKD. Male mice exhibited persistent renal dysfunction, progressive fibrosis, glomerulosclerosis, and tubular injury across all timepoints. In contrast, female mice showed partial recovery of renal function, reduced histopathological damage, and attenuated fibrosis. These findings demonstrate the temporal and structural differences between sexes and reinforce biological sex as a critical determinant of kidney disease progression.

These findings are consistent with epidemiological data showing that, although women have a higher prevalence of CKD stages 3–5, men more commonly present with albuminuria (stages 1–2), experience a faster decline in kidney function, and progress more rapidly to end-stage kidney disease. Consequently, men are overrepresented in dialysis and transplant populations [18,19]. This disparity may be partially explained by sex-specific differences in kidney hemodynamics. As summarized by van Eeghen et al. [4], men are more prone to glomerular hyperfiltration, elevated glomerular pressure, and increased filtration fraction, which constitute hemodynamic states that accelerate renal injury. In contrast, women exhibit more favorable adaptive responses to physiological stress, including preserved GFR and effective renal plasma flow, alongside lower renal vascular resistance. These protective effects may be attributed to estradiol-mediated modulation of nitric oxide bioavailability, endothelin signalling, and dampened activation of the renin-angiotensin-aldosterone system (RAAS). Notably, a prospective longitudinal study by Melsom et al. [5] using measured GFR in a healthy adult population confirmed that women have a significantly slower age-related decline in GFR compared with men, independent of baseline function or comorbidities. Such mechanisms and trajectory differences may underlie the enhanced renal resilience observed in our model, in which females maintained GFR and showed reduced fibrosis following uIRIx injury. Together, these observations reiterate the importance of applying age- and sex-specific reference values when interpreting kidney function, as baseline levels and physiological trajectories differ markedly between males and females.

These sex-specific differences in the injury response are likely driven by distinct molecular and hormonal pathways. Estrogens have been shown to enhance endothelial function [20], suppress profibrotic signalling, and reduce oxidative stress, while androgens such as testosterone promote apoptosis and inflammation via profibrotic pathways [21]. These protective effects may underlie the reduced KIM-1 expression and limited tubular dilation observed in uIRIx female kidneys. While both sexes showed elevated KIM-1 post-uIRIx, expression in males was stronger and more sustained, correlating with greater tubular dilation and extracellular matrix accumulation, indicative of progressive injury. In contrast, transient expression in females suggests more effective adaptive repair. To confirm comparable initial injury, urinary KIM-1 to creatinine ratio was used as a sensitive biomarker post-uIRI. This allowed direct comparison of sex-specific recovery responses, as KIM-1/Cr reliably detects tubular injury even in the absence of changes in eGFR or proteinuria [22]. However, previous work by Kwekel et al. reported a pronounced sex difference in KIM-1 mRNA expression during the life cycle, with female rats exhibiting >23-fold higher baseline levels than males at 8 weeks of age [23], highlighting a limitation of extrapolating male-based preclinical biomarker data to females. In our uIRIx model, IHC and ELISA analyses revealed that injured female mice exhibited lower and more transient KIM-1 protein expression compared with injured males. This may indirectly reflect female-specific protective mechanisms against renal insult, as well as dynamic post-transcriptional regulation, sex-specific differences in injury responses, and distinct repair processes. Collectively, these observations underscore the importance of considering sex and multiple biomarker readouts when evaluating kidney injury and recovery.

Consistent with the severity of tubular injury, sex-specific differences in inflammatory cell dynamics were evident following uIRIx. Both F4/80^+^ macrophages and CD3^+^ T cells increased markedly at day 14, but infiltration was more extensive and persistent in males, remaining elevated even at day 98, whereas females showed near-complete resolution by the same stage. These findings suggest a more efficient inflammatory resolution, which is a key determinant of adaptive repair rather than progression to fibrosis. This observation aligns with previous studies showing that estrogens attenuate renal inflammation by inhibiting NF-κB activation, macrophage recruitment, and Th17/Treg balance, thereby shortening the pro-inflammatory phase [14,23–25]. Conversely, testosterone has been linked to enhanced monocyte chemoattractant protein-1 and tumor necrosis factor-α expression, amplifying leukocyte infiltration and tubular apoptosis [26]. The sustained macrophage and T-cell presence in males therefore likely contributes to the maladaptive repair loop that promotes fibroblast activation and extracellular matrix deposition [27].

Sex-specific differences in vascular adaptation were detected by CD31^+^ capillary analysis. At day 14, male kidneys exhibited a transient increase in CD31^+^ density, which may be linked to the role of CD31 in leukocyte trans-endothelial migration. CD31 signalling has been shown to facilitate uropod polarization and regulate integrin-extracellular matrix detachment, thereby promoting efficient leukocyte passage across the endothelium [28]. This early vascular activation was accompanied by enhanced macrophage and T-cell infiltration as observed in our model. However, by day 98, this early male response failed to translate into sustained microvascular recovery; this may be attributed to prolonged inflammatory and oxidative stress-mediated stimulation of the endothelium, which, when persistent or excessive, drives a shift from transient activation to structural injury, leading to endothelial detachment, junctional breakdown, and loss of microvascular integrity [29]. In contrast, females demonstrated delayed but effective restoration of CD31^+^ capillaries, indicative of more efficient endothelial repair and vascular protection.

The divergence in fibrotic responses between sexes was also evident. Male uIRIx mice showed progressive interstitial accumulation of collagen I and III, peaking by day 70, whereas female mice exhibited significantly lower and delayed fibrosis throughout the study. This aligns with prior reports that estrogen signalling inhibits epithelial-to-mesenchymal transition and suppresses TGF-β1-mediated fibrotic pathways shown in other systems [11,30,31]. In our study, the temporal dissociation between injury onset and fibrosis in females suggests an active limitation of fibrotic remodelling rather than a simple delay. These findings are supported by Lima-Posada et al. [14], who used a time-course rat model of renal ischemia to demonstrate that, despite similar initial AKI, only male rats developed CKD characterized by persistent fibrosis, proteinuria, oxidative stress, and suppression of HIF1α and VEGF. In contrast, female rats were protected and exhibited early upregulation of antioxidant defenses and pro-reparative factors such as eNOS, HIF1α, and TGF-β. Notably, this protection was lost in oophorectomized females, implicating ovarian hormones in maintaining long-term renal integrity. In addition to hormonal influences, sex differences in renal transporter expression [32] and RAAS activity [33,34] may contribute to divergent outcomes following injury. For instance, higher sodium-glucose cotransporter 2 expression in female kidneys has been proposed to enhance tubular reabsorption and energy efficiency, potentially supporting recovery after nephron loss [35]. Likewise, greater activation of the classical RAAS pathway in males, including heightened responsiveness to angiotensin II, may exacerbate hemodynamic stress and fibrosis [34].

Although total glomerular number is generally lower in females due to smaller kidney size, nephron density is comparable between sexes, and these anatomical differences are not thought to drive divergent disease progression or age-related GFR decline. In our study, glomerular hypertrophy progressively developed in both male and female mice over time, in both the sham control and injured groups. This hypertrophy may represent a compensatory adaptation aimed at preserving filtration capacity in the setting of reduced nephron mass. A similar pattern has been reported in aging female rodents, including the stroke-prone spontaneously hypertensive rat model (SHRSP), where females were protected from renal and cardiac fibrosis compared with age-matched males under the same hypertensive stress [11]. Males exhibited greater glomerular hypertrophy, glomerulosclerosis, and fibrosis, accompanied by increased renal senescence markers, suggesting that early aging accelerates injury. In contrast, female SHRSP rats showed reduced scarring and senescence with age, supporting sex-specific regulation of glomerular adaptation and cellular aging as drivers of divergent disease progression [11]. Interestingly, injured males at day 14 showed a reduction in glomerular size compared with controls, which could be associated with the marked reduction in GFR observed post-nephrectomy. While unilateral nephrectomy itself can elicit sex-dependent compensatory responses [36], the patterns observed in our study, particularly the reduced glomerular size in injured males and adaptive hypertrophy in females, indicate that the sex differences in glomerular remodelling likely reflect the combined effects of ischemic injury and nephrectomy, rather than nephrectomy alone. In our model, glomerular hypertrophy in females occurred in the absence of significant glomerulosclerosis or fibrosis, indicating an adaptive, rather than maladaptive, response. Conversely, the smaller glomerular size in injured males at both early and late stages may reflect impaired compensatory remodelling or early atrophy due to unresolved injury and senescence-related dysfunction.

Collectively, this study highlights the complexity of sex-specific renal responses and supports the notion that female mice are inherently more resilient to ischemic injury and the downstream fibrotic cascade. These differences likely reflect an interplay of hormonal, molecular, and cellular mechanisms that influence not only the severity of acute injury but also the trajectory of long-term repair and fibrosis. Further investigation into sex-specific transcriptional and epigenetic regulators of tubular and glomerular adaptation will be crucial in understanding these mechanisms.

## Clinical perspectives

- **Background:** Sex differences are increasingly recognised as critical determinants of kidney disease progression, yet their role in post-ischemic recovery remains unclear.
- **Results:** Using a longitudinal uIRIx mouse model, we found that males develop sustained renal dysfunction, fibrosis, and inflammation, while females exhibit partial functional recovery and reduced structural injury.
- **Significance:** These data identify biological sex as a key modifier of renal repair and support the development of sex-specific strategies to prevent AKI-to-CKD progression.

## Supplementary Material

Supplementary Figures S1-S2

## Data Availability

No public datasets were generated or deposited as part of this study. The data supporting the findings are available from the corresponding author upon reasonable request.

## Open Access

Open access for this article was enabled by the participation of Monash University in an all-inclusive Read & Publish agreement with Portland Press and the Biochemical Society under an agreement with CAUL.

## Abbreviations

AKI: acute kidney injury
CKD: chronic kidney disease
GFR: glomerular filtration rate
IRI: ischemia-reperfusion injury
KIM-1: kidney injury molecule-1
PAS: periodic acid Schiff
PSR: picrosirius red
RAAS: renin-angiotensin-aldosterone system
s.c.: subcutaneously
SEM: standard error of the mean
SHRSP: stroke-prone spontaneously hypertensive rat
uACR: urinary albumin/creatinine ratio
uIRIx: unilateral ischemia-reperfusion model with contralateral nephrectomy
