# Supplementary material for: Sex-dependent differences in the progression of renal injury and fibrosis following ischemic acute kidney injury
Source: Clin Sci (Lond). 2026 Feb 17;140(3):275–90. doi: 10.1042/CS20250136 (PMC13108846; doi:10.1042/CS20250136)
Supplement: Supplementary Figures S1-S2 [file CS-2025-0136_supp.pdf]

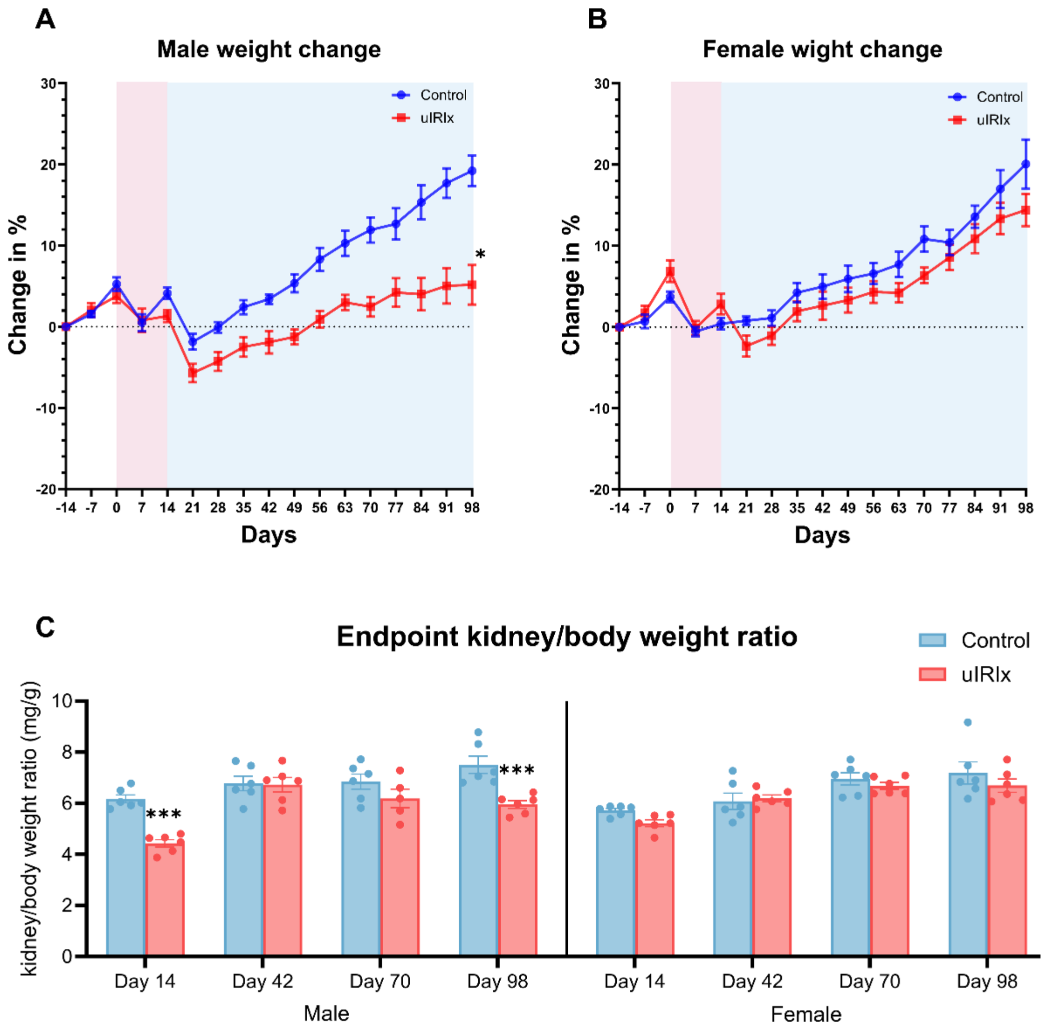

Supplementary Figure 1. Longitudinal body weight changes and endpoint kidney/body weight ratio in male and female mice. Body weight changes in males (A) and females (B) are expressed as % change from day 14 as measured weekly, kidney/body weight ratio (C) are measured at endpoint (mg/g). Data are shown as mean ± SEM at each time point. *P < 0.05 versus control within the sex at the same timepoint from day 14 to day 98; ***P < 0.01 versus control within the sex at the same timepoint.

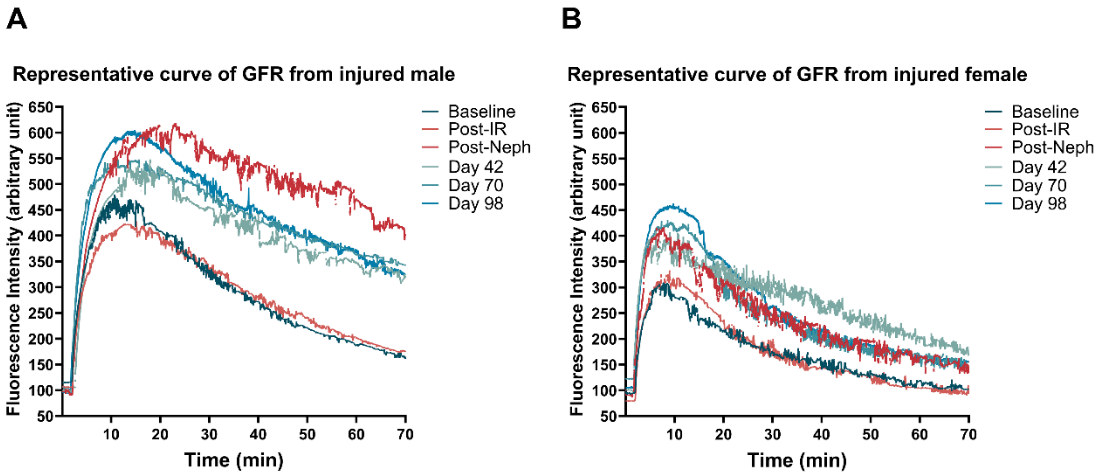

Supplementary Figure 2. Representative fluorescence clearance curves of transdermal glomerular filtration rate (GFR) in male and female mice subjected to unilateral ischemia/reperfusion injury with contralateral nephrectomy (uIRIx). Representative transdermal fluorescence intensity-time curves showing the clearance of FITC-sinistrin as a surrogate for GFR at different time points following IRI and nephrectomy in (A) male and (B) female C57BL/6 mice. Each curve represents a single mouse at baseline (pre-IRI), post-IRI (day 3), post-nephrectomy (day 17), and at 42, 70, and 98 days after IRI.

MONASH University

MONASH BIOMEDICINE DISCOVERY INSTITUTE

8th January 2026

Dear Editors,

Thank you for the opportunity to revise our manuscript. In response to Reviewer 2, we have now included kidney weight data normalised to body weight, which are presented in Supplementary Figure S1C and described in the Results.

With respect to the request to quantify Figure 1A, we wish to clarify that this panel is intended to present representative histological images. The same kidneys have already been subjected to comprehensive, blinded quantitative analyses across multiple structural domains, including tubular dilation, glomerulosclerosis, interstitial fibrosis, inflammatory cell infiltration, peritubular capillary density, and injury marker expression, all of which are reported in Figures 1D, 3, 4, and 5. We therefore considered that additional semi-quantitative scoring of the PAS images in Figure 1A would be redundant and methodologically inferior to the objective morphometric analyses already provided.

In addition, we have amended the supplementary figure file names to ensure they clearly correspond to the figures referenced in the text, and we have suggested a cover image for your consideration, as requested.

Kind regards,

Professor Sharon D. Ricardo
Monash Biomedicine Discovery Institute, Monash University

Department of Pharmacology, Monash University
9 Ancora Imparo Way, Clayton 3800 Australia
www.monash.edu/discovery-institute
CRICOS provider number 00008C ABN 12 377 614 012
